# Striped catfish (*Pangasianodon hypophthalmus*) exploit food sources across anaerobic decomposition- and primary photosynthetic production-based food chains

**DOI:** 10.1038/s41598-023-41209-y

**Published:** 2023-08-26

**Authors:** Ayano Medo, Nobuhito Ohte, Hiroki Kajitani, Takashi Nose, Yuki Manabe, Tatsuya Sugawara, Yuji Onishi, Akiko S. Goto, Keisuke Koba, Nobuaki Arai, Yasushi Mitsunaga, Manabu Kume, Hideaki Nishizawa, Daichi Kojima, Ayako Yokoyama, Toshiro Yamanaka, Thavee Viputhanumas, Hiromichi Mitamura

**Affiliations:** 1 Graduate School of Informatics, Kyoto University, Yoshida-Honmachi, Sakyo-ku, Kyoto, 606-8501 Japan; 2 Graduate School of Agriculture, Kyoto University, Kitashirakawa-Oiwake-cho, Sakyo-ku, Kyoto, 606-8502 Japan; 3 Center for Ecological Research, Kyoto University, 2-509-3 Hirano, Otsu, Shiga 520-2113 Japan; 4 Field Science Education and Research Center, Kyoto University, Kitashirakawa-Oiwake-cho, Sakyo-ku, Kyoto, 606-8502 Japan; 5 Faculty of Agriculture, Kindai University, 3327-204 Nakamachi, Nara, 631-8505 Japan; 6 Department of Ocean and Environmental Sciences, Tokyo University of Marine Science and Technology, 4-5-7 Konan, Minato-ku, Tokyo, 108-8477 Japan; 7 Inland Aquaculture Research and Development Division, Department of Fisheries, 50 Phahonyothin Rd., Lat Yao, Chatuchak, Bangkok, 10900 Thailand; 8 Present Address: Research Institute for Humanity and Nature, 457-4 Kamigamo-Motoyama, Kita-ku, Kyoto, 603-8047 Japan

**Keywords:** Freshwater ecology, Stable isotope analysis

## Abstract

Dietary information from aquatic organisms is instrumental in predicting biological interactions and understanding ecosystem functionality. In freshwater habitats, generalist fish species can access a diverse array of food sources from multiple food chains. These may include primary photosynthetic production and detritus derived from both oxic and anoxic decomposition. However, the exploitation of anoxic decomposition products by fish remains insufficiently explored. This study examines feeding habits of striped catfish (*Pangasianodon hypophthalmus*) at both adult and juvenile stages within a tropical reservoir, using stable carbon, nitrogen, and sulfur isotope ratios (*δ*^13^C, *δ*^15^N, and *δ*^34^S, respectively) and fatty acid (FA) analyses. The adult catfish exhibited higher *δ*^15^N values compared to primary consumers that feed on primary photosynthetic producers, which suggests ingestion of food sources originating from primary photosynthetic production-based food chains. On the other hand, juvenile catfish demonstrated lower *δ*^15^N values than primary consumers, correlating with low *δ*^34^S value and large proportions of bacterial FA but contained small proportions of polyunsaturated FA. This implies that juveniles utilize food sources from both anoxic decomposition and primary photosynthetic production-based food chains. Our results indicate that food chains based on anoxic decomposition can indeed contribute to the dietary sources of tropical fish species.

## Introduction

Understanding the feeding habits of aquatic animals is crucial for predicting biological interactions and comprehending ecosystem function. Freshwater ecosystems, characterized by diverse habitats and species, rely on multiple food chains that sustain complex food webs^1,2^. Algal primary production and microbial decomposition of autochthonous and allochthonous organic sources, such as detritus, serve as essential energy sources for food webs in freshwater ecosystems^3^. Furthermore, organic matter derived from autochthonous and allochthonous origins act as a substrate for anoxic decompositions by microorganisms, including methanogens^4^ and sulfate-reducing bacteria (SRB)^5,6^. The products of anoxic decomposition, such as methane and sulfide, can be transferred to higher trophic levels through chemoautotrophic microorganisms^7,8^. Generalist fish species often exploits a wide range of food sources, including phytoplankton and detritus^9^, but the contribution of anoxic decomposition products to fishes remains poorly understood.

Stable isotopes in animal tissues are widely used to determine the feeding habits and organic sources of aquatic organisms including generalist fish species. Stable carbon and nitrogen isotope ratios (*δ*^13^C and *δ*^15^N values, respectively) are particularly powerful tools for determining spatial/trophic niches and energy flows in the food webs of freshwater ecosystems^10,11^. *δ*^13^C value in consumer tissues discriminates between food webs based on planktonic (phytoplankton; *δ*^13^C =  − 32 ± 2‰) or benthic algae (periphyton; *δ*^13^C = − 27 ± 3‰)^12^. On the other hand, *δ*^15^N value is used to identify trophic positions in a food web^13–15^. However, this conventional method may limit to define detritus-based food chains regardless of oxic and anoxic decomposition, considering that *δ*^13^C values of sedimentary detritus overlap among those of phytoplankton and terrestrial organic matters^16^. Fatty acid (FA) analysis is used to define the contribution of detritus and primary photosynthetic producers to aquatic consumers^17,18^ because the FA compositions of bacteria is distinct from that of phytoplankton^19–21^, and the FA composition in consumer tissues reflects that in their diet^22,23^. Furthermore, the stable sulfur isotope ratio (*δ*^34^S value) can be used to distinguish the origin of organic matter under oxic and anoxic conditions^24^. A difference in *δ*^34^S value likely results from a low *δ*^34^S value caused by SRB activity under anoxic conditions (ca. − 20‰)^25,26^, relative to *δ*^34^S values of sulfate and organisms from oxygenated conditions (0 to + 10‰ for freshwater)^27^.

The striped catfish (*Pangasianodon hypophthalmus*), an endemic species to the Mekong and Chao Phraya rivers in Southeast Asia, is a large freshwater fish that can grow up to 130 cm in body length^28^. Due to its popularity aquaculture, the catfish has been introduced into tropical inland waters in Southeast Asia^28^, South Asia^29^, and South America^30^. Often, released fish settle in the natural environments of these areas. Numerous studies have investigated aquaculture and physiological aspects of striped catfish using fingerlings^31,32^. In the Mekong River, the catfish can be found in various habitats, including mainstreams, floodplain, and lakes^33^. Annual migration patterns likely differ between juvenile and adult striped catfish^33^, potentially leading to variations in food sources across different growth stages. The catfish are considered generalists, feeding omnivorously on algae and detritus, owing to their relative gut length^34^. Furthermore, stomach content analysis has revealed the presence of phytoplankton, detritus, and zooplankton in their diets^35^. However, their trophic positions within food webs remain unclear.

**Figure 1:**
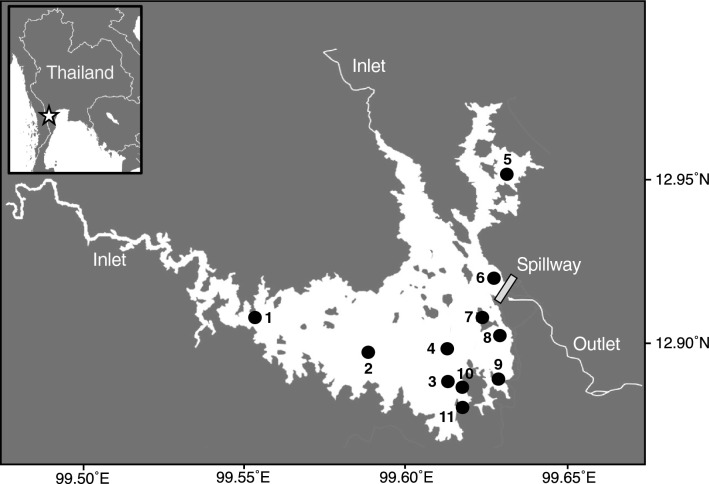
Map of the Kaeng Krachan Reservoir, Thailand. Closed circles represent the sampling sites. Bottom sediments were collected at sites 1–7 and 9–11. Water samples were collected at sites 1–8. Physico-chemical profiles were measured at sites 1–6.

This study aims to investigate the feeding habits of juvenile and adult striped catfish inhabiting the Kaeng Krachan Reservoir, Thailand (Fig. 1). In the reservoir, hatchery-reared striped catfish measuring 10 cm or less in total length (TL) has been released for fishery stock enhancement, and these fish have grown to reach 100 cm in TL. Due to fishery stock management practices, catfish reaching 30 cm or more in TL have been captured in the reservoir. Consequently, our investigation focused on juvenile (36–50 cm in TL) and adult (80–106 cm in TL). To assess the contribution of primary photosynthetic production and sedimentary detritus-based food chains, including oxic and anoxic decomposition, to their food sources, we analyzed three isotope ratios (*δ*^13^C, *δ*^15^N, and *δ*^34^S) and FA biomarkers in the tissues of juvenile and adult catfish. Additionally, we inferred the trophic positions of striped catfish within primary photosynthetic production-based food chains by comparing their *δ*^13^C and *δ*^15^N values with those of primary consumers that feed on primary photosynthetic producers and other sympatric fish species with well-documented feeding habits. Furthermore, we examined *δ*^34^S values in sulfate collected from water and sulfide obtained from sediment to establish the baselines for oxic and anoxic decomposition in the reservoir.

## Results

### Aqueous and sediment geochemistry

Water temperature (WT), chlorophyll *a* fluorescence, and dissolved oxygen (DO) were measured in the Kaeng Krachan Reservoir in September 2019. In the reservoir, WT and chlorophyll *a* fluorescence declined with increasing depth from the surface at all sampling sites. A thermocline was observed at approximately water depths of 5–10 m (sites 1–6; Figs. 1 and S1). We also observed the boundaries of DO concentrations at water depths of 5–10 m. The DO concentration was higher in the surface zone than in the profundal zone, but increased again near the reservoir bottom at all sites except for site 5 (Fig. S1). In September 2019, we also measured the sulfate ion concentration and *δ*^34^S values of sulfate ions (*δ*^34^S_sulfate_) in water. The sulfate concentration was 8.4–53.8 µM and declined with increasing depth from the surface at all sampling sites. The *δ*^34^S_sulfate_ value was + 9.0‰ to + 12.3‰ (Fig. S2). Sediment samples were collected in September 2019 to analyze the *δ*^34^S values of acid-volatile sulfide (AVS) and total sulfur (TS) (*δ*^34^S_AVS_ and *δ*^34^S_TS_, respectively). TS and AVS were detected in sediments on the sites below water depths of 15.5 m (sites 2–6; Table S1). At all sites, *δ*^34^S_AVS_ values (range + 6.3‰ to + 9.6‰) were intermediate between *δ*^34^S_sulfate_ and *δ*^34^S_TS_ values (range + 6.0‰ to + 8.4‰) (Table S1).

### Stable isotope signatures of food web components in the Kaeng Krachan Reservoir

**Figure 2:**
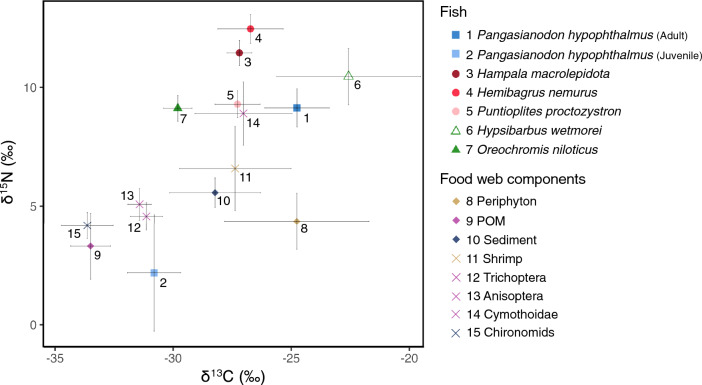
Stable carbon and nitrogen isotope values (*δ*^13^C and *δ*^15^N) of all samples collected from the Kaeng Krachan Reservoir. Points and error bars indicate the mean and standard deviation of stable isotope ratios. POM indicates particulate organic matter. All data except for the striped catfish (*Pangasianodon hypophthalmus*) were cited from Medo et al., 2021^41^.

In the reservoir, we collected 25 juvenile striped catfish in August–September and November–December 2019, as well as 11 adult striped catfish in November–December 2019. The adult striped catfish exhibited higher values for three isotope signatures (*δ*^13^C = − 24.8 ± 1.4 [mean ± SD] ‰, *δ*^15^N = 9.1 ± 0.8‰, *δ*^34^S = 6.2 ± 0.9‰) compared to the juvenile catfish (*δ*^13^C = − 30.8 ± 1.1‰, *δ*^15^N = 2.2 ± 2.5‰, *δ*^34^S = 0.1 ± 2.0‰) (Table 1). We compared the *δ*^13^C and *δ*^15^N values among the catfish and other sympatric fish species collected in August–September and November–December 2019, which included the piscivore *Hampala macrolepidota*^36^, carnivore *Hemibagrus nemurus*^37^, insectivore *Puntioplites proctozystron*^38^, herbivore *Hypsibarbus wetmorei*^39^, and herbivore *Oreochromis niloticus*^40^ (cited from Medo et al.^41^). The Steel–Dwass test showed that the *δ*^13^C value of adult catfish did not differ from that of *H. nemurus* (*δ*^13^C = − 26.7 ± 1.4‰, *p* = 0.05) and *H. wetmorei* (*δ*^13^C = − 22.6 ± 3.0‰, *p* = 0.63), but was significantly higher than that of *H. macrolepidota* (*δ*^13^C = − 27.2 ± 0.5‰, *p* = 0.01), *P. proctozystron* (*δ*^13^C = − 27.3 ± 1.0‰, *p* = 0.02), and *O. niloticus* (*δ*^13^C = − 29.8 ± 0.6‰, *p* = 0.002). The Steel–Dwass test showed that the *δ*^13^C value of juvenile catfish was significantly lower than that of *H. macrolepidota* (*p* = 1.0e^−4^), *H. nemurus* (*p* = 4.5e^−6^), *P. proctozystron* (*p* = 1.0e^−4^), and *H. wetmorei* (*p* = 4.8e^−5^), but did not differ from that of *O. niloticus* (*p* = 0.22). The Steel–Dwass test showed that the *δ*^15^N value of adult catfish did not differ from that of *P. proctozystron* (*δ*^15^N = 9.3 ± 0.6‰, *p* = 1.00), *H. wetmorei* (*δ*^15^N = 10.5 ± 1.2‰, *p* = 0.18), and *O. niloticus* (*δ*^15^N = 9.1 ± 0.5‰, *p* = 1.00), but was significantly lower than that of *H. macrolepidota* (*δ*^15^N = 11.5 ± 0.5‰, *p* = 0.002) and *H. nemurus* (*δ*^15^N = 12.5 ± 0.6‰, *p* = 4.0e^−4^). The Steel–Dwass test showed that the *δ*^15^N value of juvenile catfish was significantly lower than that of *H. macrolepidota* (*p* = 1.0e^−4^), *H. nemurus* (*p* = 3.4e^−6^), *P. proctozystron* (*p* = 1.0e^−4^), *H. wetmorei* (*p* = 1.0e^−4^), and *O. niloticus* (*p* = 4.8e^−5^). We compared the *δ*^15^N values between juvenile catfish and primary consumers that feed on primary photosynthetic producers, Trichoptera (*n* = 14), collected in September and November 2019 (cited from Medo et al.^41^). The *δ*^15^N value of juvenile catfish was lower compared to Trichoptera (*δ*^15^N = 4.6 ± 0.6‰) (Table 1; Fig. 2). Furthermore, we compared the *δ*^15^N values among juvenile catfish and basal food sources, including periphyton (*n* = 26), particulate organic matter (POM; *n* = 48), and lake bottom sediments (*n* = 16), collected in September and November 2019 (cited from Medo et al.^41^). The *δ*^15^N value of juvenile catfish was lower than that of the basal sources (*δ*^15^N = 3.3 ± 1.4‰, 4.4 ± 1.2‰, and 5.6 ± 0.6‰ for POM, periphyton, and sediments) (Table 1; Fig. 2). We did not observe any horizontal and vertical variations in the *δ*^13^C and *δ*^15^N values in POM and sediments (Fig. S3), nor did we detect large seasonal variations in the *δ*^13^C and *δ*^15^N values in the food web components between August–September and November–December 2019 (Fig. S4).

**Table 1:** Mean ± SD of stable isotope ratios in carbon (δ^13^C), nitrogen (δ^15^N), and sulfur (δ^34^S) for samples collected in the Kaeng Krachan Reservoir in August–September and November–December 2019.

	*N*	δ^13^C (‰)	δ^15^N (‰)	δ^34^S (‰)	Total length (cm)	Feeding habits
Fish species						
*Pangasianodon hypophthalmus* (Adult)	11	− 24.8 ± 1.4	+ 9.1 ± 0.8	+ 6.2 ± 0.9	80–106	–
*Pangasianodon hypophthalmus* (Juvenile)	25	− 30.8 ± 1.1	+ 2.2 ± 2.5	+ 0.1 ± 2.0	34–50	–
*Hampala macrolepidota*	10	− 27.2 ± 0.5	+ 11.5 ± 0.5	–	25–40	Piscivore^36^
*Hemibagrus nemurus*	15	− 26.7 ± 1.4	+ 12.5 ± 0.6	–	20–70	Carnivore^37^
*Puntioplites proctozystron*	10	− 27.3 ± 1.0	+ 9.3 ± 0.6	–	20–30	Insectivore^38^
*Hypsibarbus wetmorei*	11	− 22.6 ± 3.0	+ 10.5 ± 1.2	–	25–35	Herbivore^39^
*Oreochromis niloticus*	10	− 29.8 ± 0.6	+ 9.1 ± 0.5	–	10–50	Herbivore^40^
Others						
Periphyton	26	− 24.8 ± 3.1	+ 4.4 ± 1.2	–		
POM	48	− 33.5 ± 0.8	+ 3.3 ± 1.4	–		
Sediment	16	− 28.2 ± 1.9	+ 5.6 ± 0.6	–		
Shrimp	7	− 27.4 ± 2.4	+ 6.6 ± 1.8	–		
Trichoptera	14	− 31.1 ± 0.7	+ 4.6 ± 0.6	–		
Anisoptera	18	− 31.4 ± 0.5	+ 5.1 ± 0.7	–		
Cymothoidae	9	− 27 ± 2.1	+ 8.9 ± 1.3	–		
Chironomids	24	− 33.6 ± 1.1	+ 4.2 ± 0.6	–		

The stable carbon and nitrogen isotope ratio data for fish species and other food web components other than the striped catfish (*Pangasianodon hypophthalmus*) were cited from Medo et al.^41^. Adult striped catfish was collected only in November–December 2019.

### Fatty acid compositions of the striped catfish

**Figure 3:**
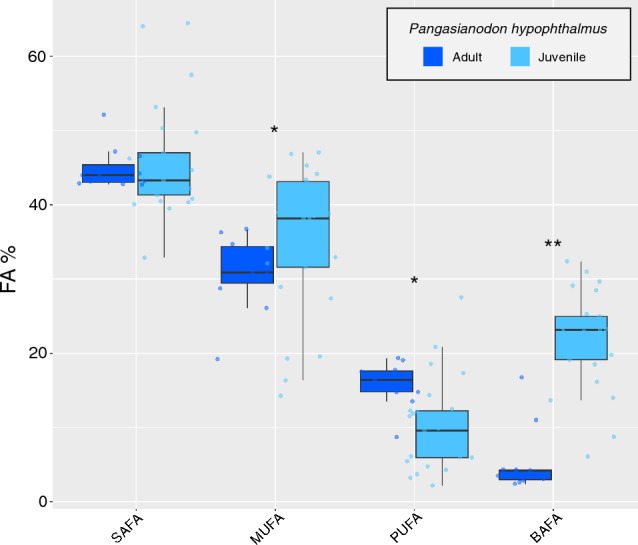
Composition of fatty acid (FA) groups (% relative to total FA) in adult and juvenile in the striped catfish (*Pangasianodon hypophthalus*). Asterisk indicates a significantly difference at **p < 0.001; *p < 0.05.

The most abundant FA was saturated FA (SAFA) in adult and juvenile catfish (44.8 ± 2.9% and 45.8 ± 7.4%, respectively; Table 2). The second dominating FA in the adult and juvenile catfish was monounsaturated FA (MUFA) (31.0 ± 5.1% and 35.1 ± 9.6%, respectively). Bacterial FA (BAFA) and MUFA were significantly higher in juvenile catfish than that in adult catfish (Wilcoxon rank-sum test*, p* = 1.2e^−7^ and 0.04, respectively) (Fig. 3). The abundance of i- and a- branched FAs, as well as methane-oxidizing bacteria (MOB) specific FAs^19^, was less than 1% of all FAs (Table 1). Polyunsaturated FA (PUFA) was significantly higher in adult catfish than in juvenile catfish (Wilcoxon rank-sum test, *p* = 0.001) (Fig. 3).

**Table 2:** Fatty acid (FA) compositions (% relative to total FA, mean ± SD) of the striped catfish (*Pangasianodon hypophthalmus*) in the Kaeng Krachan Reservoir.

Fatty acids	Adult	Juvenile
	*n* = 11	*n* = 25
14:0	4.1 ± 0.6	4.6 ± 1.4
i-15:0	0.3 ± 0.2	0.6 ± 0.3
a-15:0	0.1 ± 0.1	0.3 ± 0.1
15:0	0.5 ± 0.2	1.9 ± 1.0
16:0	30.0 ± 1.8	26.9 ± 2.6
16:1n-7	1.6 ± 0.4	11.5 ± 4.0
i-17:0	0.3 ± 0.1	0.5 ± 0.2
17:0	0.6 ± 0.2	1.6 ± 0.7
17:1	0.2 ± 0.1	0.5 ± 0.1
18:0	7.7 ± 0.6	6.6 ± 1.0
18:1n-9	25.1 ± 5.5	12.9 ± 4.0
18:1n-7	3.0 ± 4.6	9.0 ± 2.9
18:2n-6	1.3 ± 0.3	1.4 ± 0.7
18:3n-6	0.2 ± 0.2	0.2 ± 0.1
18:3n-3	1.8 ± 0.4	1.3 ± 1.2
18:4n-3	1.1 ± 0.2	0.3 ± 0.3
20:0	0.1 ± 0.0	0.2 ± 0.1
20:1n-9	1.1 ± 0.2	1.1 ± 0.4
20:2n-6	0.1 ± 0.1	0.3 ± 0.1
20:3n-6	0.4 ± 0.1	0.5 ± 0.3
20:4n-6	0.6 ± 0.3	1.9 ± 1.3
20:3n-3	0.6 ± 0.4	0.2 ± 0.1
20:4n-3	2.0 ± 0.6	0.6 ± 0.5
20:5n-3	2.4 ± 0.4	1.3 ± 0.7
22:6n-3	5.3 ± 1.9	2.1 ± 1.9
24:0	0.4 ± 0.8	0.1 ± 0.2
24:1n-9	0.0 ± 0.0	0.1 ± 0.1
SAFA	44.8 ± 2.9	45.8 ± 7.4
MUFA	31.0 ± 5.1	35.1 ± 9.6
PUFA	15.8 ± 3.0	10.1 ± 6.1
BAFA	5.4 ± 4.4	21.9 ± 6.5

Bacterial FA (BAFA) included 15:0, 17:0 and its branched iso- and anteiso-homologues, 16:1n-7, and 18:1n-7, which is widely observed in bacteria^69,70^.

*SAFA* sum of saturated fatty acids, *MUFA* sum of monounsaturated fatty acids, *PUFA* sum of polyunsaturated fatty acids.

### Relationships among stable isotope signatures and fatty acid compositions

**Figure 4:**
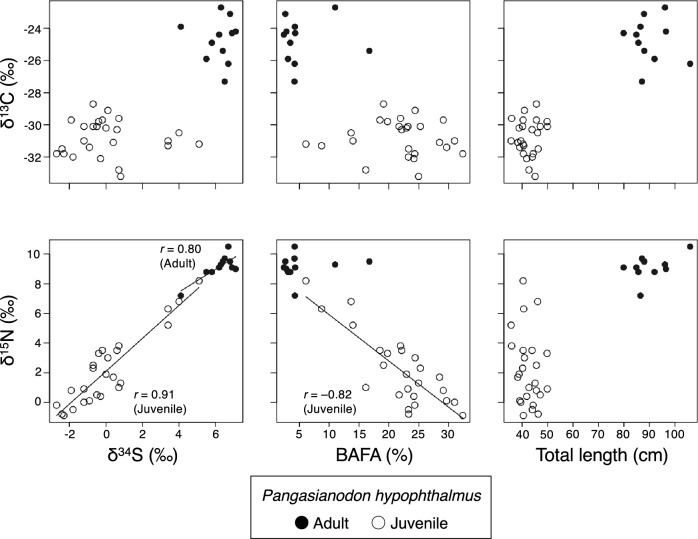
Correlations among three stable isotope ratios (carbon [*δ*^13^C], nitrogen [*δ*^15^N], and sulfur [*δ*^34^S]), bacterial fatty acid (BAFA), and total length (cm) in the striped catfish (*Pangasianodon hypophthalmus*). Solid lines indicate significant correlations at Pearson's correlation coefficient (*p* < 0.05).

There was a significant correlation between *δ*^15^N and *δ*^34^S in adult catfish (Pearson's correlation coefficient [*r*] = 0.80, *p* = 0.003). Likewise, there were significant correlations between *δ*^15^N and *δ*^34^S (*r* = 0.91, *p* = 2.0e^−10^), and *δ*^15^N and BAFA (*r* = − 0.82, *p* = 4.5e^−7^), for juvenile catfish (Fig. 4).

## Discussion

This study indicates that the striped catfish exploited food sources across multiple food chains, ranging from anoxic decomposition by bacteria to primary photosynthetic production by algae. The stable isotope signatures and FA compositions were distinct between juvenile and adult catfish. Our *δ*^13^C and *δ*^15^N analyses indicated that the primary producers contributed to the food sources for adult catfish but not entirely for juvenile catfish. The lower *δ*^15^N values relative to those of primary consumers in juvenile catfish were associated with low *δ*^34^S values and high proportions of BAFA, indicating the contribution of anoxic decomposition-based food chains to their diet. Although striped catfish were previously thought to feed omnivorously on phytoplankton, detritus, and zooplankton^32^, this study suggests that the feeding habits of catfish differ between adults and juveniles.

Since the *δ*^15^N value of adult catfish did not significantly differ from that of the herbivore *O. niloticus*^40^ and insectivore *P. proctozystron*^38^, it is likely that adult catfish were primary to secondary consumers in primary photosynthetic production-based food chains. The FA composition of adult catfish was characterized by high proportions of PUFA, which are synthesized by phytoplankton such as diatoms, dinoflagellates, and cryptophytes, in freshwater ecosystems^22,23^. In addition to algal sources, long-chain PUFA can also be obtained from freshwater zooplankton owing to their selective accumulation^42^. Thus, adult catfish may ingest zooplankton as well as algae.

Low *δ*^15^N values in juvenile catfish suggests that they exploited food sources from anoxic decomposition-based food chains. Given a trophic discrimination factor of ca. + 3‰ between a prey item and consumer^43^, the *δ*^15^N values of consumers on primary photosynthetic production-based food chains should be higher than those of primary producers, such as periphyton and POM (*δ*^15^N = ca. + 4‰). Although primary consumers that feed on periphyton and phytoplankton can serve as an isotopic baseline for primary photosynthetic production-based food chains^10^, the *δ*^15^N values of primary consumers, Trichoptera, did not cover those observed in juvenile catfish. The decrease in *δ*^15^N values with increasing BAFA proportions and decreasing *δ*^34^S values in juvenile catfish indicates that the low *δ*^15^N values were caused by bacterial activity under anoxic conditions. Extremely low *δ*^15^N values have been observed in methanotrophic and/or chemoautotrophic communities in freshwater^44,45^, caves^46^, and the deep sea^47^, which likely results from extensive nitrogen isotope fractionation during the assimilation of dissolved inorganic nitrogen (i.e., ammonium and nitrate) by bacteria under anoxic conditions. However, despite the high proportion of BAFA, juvenile catfish also contained PUFA that are typically absent in bacteria^19,48^. Therefore, it is likely that juvenile catfish also exploited food sources from primary photosynthetic production-based food chains.

The *δ*^13^C values of juvenile catfish were the lowest among fish but were similar values to those of primary consumers, Trichoptera. Furthermore, specific FAs to MOB, such as 16:1n-8 and 18:1n-8^19^, were not detected in the tissues of juvenile catfish. These findings suggest limited contributions of methanogens to the diet of juvenile catfish. In freshwater ecosystems, phytoplankton exhibits *δ*^13^C values in the range of − 35 to − 25‰^49^, which was consistent with our results of POM. Because biogenic methane shows extremely low *δ*^13^C values (*δ*^13^C_methane_ = − 110 to − 50‰)^50^, it is thought that low *δ*^13^C values in consumer tissues are evidence that consumers incorporate methane-derived carbon via methane-oxidizing bacteria^4,7,8^. Alternatively, chemosynthetic bacteria such as sulfur- and ammonia-oxidizing bacteria can also be a low *δ*^13^C source (*δ*^13^C > − 40‰; e.g.,^51^). In contrast, Sanseverino et al.^52^ reported that MOB-specific FAs were found in freshwater fishes despite a relatively high *δ*^13^C value in tissues (*δ*^13^C = − 37 to –28‰), which suggests consumers that obtained the compounds from anoxic conditions do not necessarily present extremely low *δ*^13^C values if they obtained the compounds from anoxic conditions. It is possible that we overlooked the bacterial contributions to high trophic levels using bulk *δ*^13^C analysis alone.

According to previous reports, ingestions of anoxic decomposition-derived sources, evidenced by low *δ*^13^C values, could be associated with fishes (i) feeding on detritus^52^ and/or (ii) feeding on bacterivorous organisms such as zooplankton and chironomid larvae^53,54^. Moreover, BAFAs have been found in detritivores^55^ and benthivores^18^. Given the results of gut morphometry for adult and juvenile striped catfish^34^, it seems that the main food sources for striped catfish in both growth stages was detritus rather than animal sources. These findings indicate that juvenile catfish ingested detritus at the bottom of the reservoir to obtain anoxic decomposition-derived sources. Exploiting such organic sources has been observed in chironomid larvae and fish with high hypoxia-tolerance^4^, which is consistent with the physiological traits of striped catfish, known for their elevated hypoxia tolerance^56^.

Ontogenetic dietary shifts are widely observed in fish species and are probably driven by several factors, such as habitat use, morphological constraints, and swimming ability^57^. Our results on the differences of stable isotopes and FA between adult and juvenile catfish can be linked to habitat use at each growth stage, and perhaps juvenile access to lentic systems in the reservoir. Annual migration patterns are likely different between juvenile and adult striped catfish; juveniles spend time in the nursery and feeding grounds of the floodplain, while adults migrate between feeding and spawning grounds of the mainstream^33^. The *δ*^13^C and *δ*^34^S values of basal food sources often vary depending on the habitats, even within a lake^12,15^. Low *δ*^13^C and *δ*^34^S values, which suggest that exploitations of anoxic decomposition-derived compounds by aquatic consumers, is typically observed in restricted sites, such as the profundal zones of lakes and in the vegetation area of shallow lakes and rivers^7,8,24,58^. Although horizontal and vertical variations in *δ*^13^C and *δ*^34^S values were slight in basal sources at the reservoir, sulfide (i.e., AVS), which is an indicator of anoxic conditions, was found at restricted sites, including lentic areas and profundal zone of the reservoir.

In this study, we did not observe low *δ*^15^N values in sediments, like those found in juvenile catfish. Our results also indicated that *δ*^34^S_TS_ extracted from the sediments did not reflect the *δ*^34^S value of the organic sources of juvenile catfish. The inconsistency in stable isotope values between deposit feeders and sediments can be explained by the selection of diet particles from sedimentary organic matter^59^. Although there was no direct evidence of potential organic sources for juvenile catfish, low stable isotope ratios in catfish compared to bulk sediments suggests selective assimilation of organic matter from bulk sediments.

However, it is important to acknowledge the limitations of this study. Tropical fishes in Southeast Asia exhibit seasonal dietary shifts^60^, which were not considered in this study due to sample collections lacking seasonality. Therefore, our dietary analyses may not fully capture the feeding habits of striped catfish. Additionally, the *δ*^13^C and *δ*^15^N values in food web components, especially in short-lived organisms, are highly variable depending on the season and month^61^. Due to sample size constraints, we could not account for seasonal variations between September and November. Consequently, stable isotope data from these two months were combined into a single dataset, potentially overlooking seasonal fluctuations in food web baselines and the feeding habits of catfish during that period. Finally, it is crucial to consider that trophic discrimination factors and turnover rates of stable isotopes in animal tissues can vary depending on body size and age^62,63^. Future analyses that encompass there parameters will offer a more comprehensive understanding of the differences in feeding habits between adult and juvenile catfish, surpassing the limitations of solely utilizing their bulk stable isotope data.

Overall, stable isotope and FA composition analyses indicated that the striped catfish ingested food sources derived from anoxic decomposition and primary photosynthetic production-based food chains during juvenile stages. Meanwhile, the striped catfish exploited food sources derived from primary photosynthetic production-based food chains during adult stages. While the feeding habits of freshwater fishes are commonly described based on primary photosynthetic production and oxic decomposition-based food chains, our research demonstrated that anoxic decomposition-based food chains also contribute to the food sources of tropical freshwater fishes. Nonetheless, it remains unclear how exploiting the food sources derived from anoxic decompositions benefits fish growth and survival. Foraging at sites where dissolved oxygen is depleted may mitigate resource competitions and provide refuge areas from predators, whereas ingesting sedimentary organic matter, including bacteria, can have negative effects on somatic growth and reproduction owing to a poor-quality food^3,64^. To test these hypotheses, future studies should investigate habitat use and trade-offs between food quality and quantity.

## Methods

### Ethics statement

All experiments and sample collection described in this paper agreed with the policies of the Animal Experiment Committee of Graduate School of Informatics, Kyoto University. All fieldwork and sample processing were conducted under the permission of the National Research Council of Thailand. All fish used in this study were caught and sold by fishermen and fish markets under the permission of the Inland Fisheries Patrol Unit, Kaeng Krachan District, Department of Fisheries, Thailand. All fish tissues used in our experiments were purchased from the fish markets, and the fish were already dead when we purchased them. We did not kill the fishes ourselves.

### Study site

This study was conducted during a monomictic lake, the Kaeng Krachan Reservoir, Phetchaburi Province, western Thailand (12° 54ʹ N, 99° 36ʹ E; Fig. 1). The Kaeng Krachan Dam impounds the Phetchaburi River, which originated in Myanmar in 1966. The climate in this area can be divided into two seasons: the wet (May–October) and the dry seasons (November–April). The annual maximum depth of the reservoir is approximately 45 m during the wet season. A thermocline in the reservoir was observed at a depth of 5–15 m from April to November and an annual overturn occurred in December^65^. The productivity of the reservoir is classified as mesotrophic during the dry season and eutrophic during the wet season^66^. The Department of Fisheries, Thailand, has released hatchery-reared striped catfish < 10 cm in TL into the reservoir for fishery stock enhancement. In addition to released catfish, there is recruitment from the natural reproduction of stocked fish in the reservoir. The Department of Fisheries, Thailand imposes fishing restrictions on the striped catfish during the spawning seasons of this species, the middle of May to the middle of August. Furthermore, it is unlikely for fishermen in the reservoir to target the catfish below 30 cm in TL.

### Sample collection

Sample were collected from the end of August to the beginning of September (the end of wet season) and from the end of November to the beginning of December (the beginning of dry season) in 2019. All striped catfish used in this study were caught by a local fisherman with permission from the Inland Fisheries Patrol Unit, Kaeng Krachan District, Department of Fisheries, Thailand. We used 36 individuals of the striped catfish (36–106 cm in TL). Based on the mature size of the catfish (> 54 cm in body length)^28^, we divided them into juveniles (*n* = 25; 36–50 cm in TL) and adults (*n* = 11; 80–106 cm in TL). Consequently, 25 juvenile catfish were collected in August–September and November–December 2019, while 11 adult catfish were collected in November–December 2019. Catfish samples were collected from the dorsal and ventral muscles for stable isotope and FA analyses, respectively. In September 2019, sediment samples were collected at a few centimeters of depth, using an Ekman-Birge grab at depths of 3–30 m at ten sites (sites 1–7 and 9–11; Fig. 1). Water samples were collected in September 2019 using a Van Dorn water sampler (water capacity, 6.0 L: Miyamoto Riken Ind. Co, Ltd., Osaka, Japan) at four depths (0, 5, 10, and 2 m above the bottom of the reservoir) and seven sites (sites 1–7; Fig. 1). All samples were stored in a freezer at − 20 °C until further processing.

### Physico-chemical analysis

The WT (°C), DO (mg L^−1^), and chlorophyll *a* fluorescence (µg L^−1^) were measured at six sites (sites 1–6; Fig. 1) during September 2019. The data were observed using a conductivity-temperature-depth instrument (AAQ-RINKO, AAQ170; JFE-Advantech, Kobe, Japan). For ion concentration analysis, the water samples were vacuum filtered through a glass microfiber GF/F filter (0.7 µm mesh or nominal pore size; Whatman, Maidstone, UK) and then through a PTFE syringe filter (0.45 µm pore size; RephiLe Bioscience, Ltd., Boston, USA). We measured the sulfate ion concentration of the water column (µM) using an ion chromatography system (Dionex ICS-1100, Thermo Fisher Scientific, MA, USA) at Kyoto University.

### Stable isotope analysis

Samples of the striped catfish were oven-dried at 50 °C for 48–72 h, then crushed by scissors. The crushed samples were delipidated using a chloroform–methanol solution (2:1 v/v). The TS in the sediment samples was converted to sulfate by heating from 80 to 120 °C in a 35.5% hydrogen peroxide solution. The AVS of the sediments was liberated by anaerobic acidification in 47% concentrated sulfuric acid during active distillation (pure nitrogen gas). The liberated H_2_S was collected as a CdS precipitate in traps containing 0.5 M cadmium acetate solution. The yellow CdS precipitate was oxidized with a few drops of hydrogen peroxide solution (35.5%). These resulting sulfates derived from TS and AVS were recovered as a BaSO_4_ precipitate by adding a 0.5 M BaCl_2_ solution. The resulting BaSO_4_ precipitate and dried sediment fraction were precisely weighted, and the TS and AVS concentration was calculated in μmol-S g^−1^ of dry sediment. Sulfate in water was also recovered as a BaSO_4_ precipitate after removing major cations using a cation-exchange resin.

*δ*^13^C and *δ*^15^N values of the striped catfish were measured using EA-IRMS (Delta V equipped with an elemental analyzer Flash EA 2000, Thermo Fisher Scientific, MA, USA) in the Center for Ecological Research, Kyoto University (CERKU). *δ*^34^S value of the striped catfish and inorganic samples (i.e., BaSO_4_ from the sulfate of the water column and sulfide of the sediment) were measured using an EA-IRMS (Delta V equipped with an elemental analyzer Flash EA 2000, Thermo Fisher Scientific, MA, USA at the Research Institute for Humanity and Nature [RIHN] and IsoPrime EA, GV Instruments, Manchester, UK, at the Tokyo University of Marine Science and Technology [TUMSAT], respectively). All samples for the *δ*^34^S were mixed with excess V_2_O_5_.

The *δ*^13^C, *δ*^15^N, and *δ*^34^S (in ‰) were expressed as the deviation from standards according to the following equation:

$$\delta {\text{X }} = \, \left[ { \, \left( {{\text{R}}_{{{\text{sample}}}} /{\text{ R}}_{{{\text{standard}}}} } \right) \, - { 1}} \right] \times {1}000$$

where X is ^13^C, ^15^N, or ^34^S; R_sample_ is the ratio (^13^C/^12^C, ^15^N/^14^N or ^34^S/^32^S) in the sample; and R_standard_ is the ratio in the standard. The standard reference materials were PDB carbonate, atmospheric N_2_, and Vienna Canyon Diablo Troilite (VCDT) for the carbon, nitrogen, and sulfur samples, respectively. We calibrated the *δ*^13^C and *δ*^15^N values using three working standards (CERKU-02, 03, and 05^67^) and calibrated the *δ*^34^S value using five working standards (IAEA-S-1, IAEA-S-2, and IAEA-S-3 at RIHN, and MSS-2 and MSS-3^68^ at TUMSAT). The overall analytical precision (standard deviation, SD) for our analyses based on working standards was within ± 0.1 and ± 0.2‰ for *δ*^13^C and *δ*^15^N in CERKU, respectively, and within ± 0.3‰ for *δ*^34^S in RIHN and TUMSAT.

### Fatty acid analysis

All catfish muscle samples were freeze-dried for 24–48 h and crushed using scissors. We used 100 mg of crushed sample (dry mass) for lipid extraction using the Bligh-Dyer method. We then conducted saponification and methylation of the free lipids using HCl–methanol (one-step method). ﻿The FAME composition was quantitatively analyzed using gas chromatography (GC) coupled with a flame ionization detector (FID; GC-14B, Shimadzu Scientific Instruments, Kyoto, Japan). The structures of the individual FAMEs were separately identified by using GC with a mass spectrometer (MS; GCMS-QP5050, Shimadzu Scientific Instruments, Kyoto, Japan) and scanning electron impact mass spectra. Each piece of equipment had an Omegawax capillary column (30 m, 0.25 mm, i.d., 0.25-µm film thickness, Supelco, USA). The following temperature gradient and instrument settings were used: the GC columns initial temperature was 140 °C; this was held for 2.5 min then ramped at 4 °C/min to 240 °C and then kept for 15 min, giving a total runtime of 42.5 min. Nitrogen and helium gases were used as carriers for FID and MS, respectively. Individual FAMEs were identified by comparing their retention times with those of authentic standard mixtures, Supelco FAME mix 37 components, Supelco Bacterial Acid Methyl Ester Mix (Sigma-Aldrich, St. Louis, MO, US), and with spectrographic patterns from the mass spectral library (NIST107.LIB and NIST21.LIB). The proportion of fatty acids (%) was determined as the ratio of each FAME peak area to the total areas of all the peaks in the FID analysis. The FAs were abbreviated following the standard nomenclature X:Yn-Z, where X is the number of carbons, Y is the number of double bonds, and Z is the position of the first double bond counted from the end of the methyl group in the FA (e.g. 20:5n-3). We summarized the FA groups including the sum of SAFA, MUFA, PUFA, and BAFA. Here, BAFA included 15:0, 17:0, iso-, and anteiso- (i- and a-) branched FAs (i.e., i-15:0, a-15:0, i-17:0, and a-17:0), 16:1n-7, and 18:1n-7, as these FAs are widely observed in bacteria^69,70^.

### Statistical analysis

We used *δ*^13^C and *δ*^15^N values that were measured from several organisms in our previous study^41^; five fish species including piscivore *Hampala macrolepidota*^36^, carnivore *Hemibagrus nemurus*^37^, insectivore *Puntioplites proctozystron*^38^, herbivore *Hypsibarbus wetmorei*^39^, and herbivore *Oreochromis niloticus*^40^; other organisms and substrates were periphyton (*n* = 26), POM (*n* = 48), lake bottom sediments (*n* = 16), chironomids larvae (*n* = 24), aquatic insects (including Trichoptera, Anisoptera larvae, and Cymothoidae, *n* = 41), and shrimp (*n* = 7) (Table 1). We considered that POM represented the *δ*^13^C and *δ*^15^N values of phytoplankton in the reservoir. The POM was collected by vacuum filtering water samples through a glass microfiber GF/F filter (0.7 µm mesh or nominal pore size; Whatman, Maidstone, UK) after the water samples were collected using a Van Dorn water sampler (water capacity, 6.0 L: Miyamoto Riken Ind. Co, Ltd., Osaka, Japan)^41^. We collected the aquatic insects hiding in periphyton. All samples were collected during August–September and November–December 2019.

All statistical analyses were performed using the R software ver. 4.0.1^71^ at a statistical significance level of 0.05. Using Steel–Dwass test (*pSDCFlig* function in *NSM3* package^72^), we compared *δ*^13^C and *δ*^15^N values among fish species (i.e., the juvenile and adult catfish and other fishes). We investigated the correlations in *δ*^13^C and *δ*^15^N between three factors: (i) *δ*^34^S, (ii) BAFA, and (iii) TL using Pearson's correlation analyses (*cor.test* function).

### Supplementary Information

Supplementary Information.

## Data Availability

The datasets used and/or analyzed during the current study are available from the corresponding author on reasonable request.
