# Supplementary material for: Striped catfish (Pangasianodon hypophthalmus) exploit food sources across anaerobic decomposition- and primary photosynthetic production-based food chains
Source: Sci Rep. 2023 Aug 26;13:13992. doi: 10.1038/s41598-023-41209-y (PMC10460403; doi:10.1038/s41598-023-41209-y)
Supplement: Supplementary file 1 — Supplementary Information. [file 41598_2023_41209_MOESM1_ESM.pdf]

Supplementary Information

Striped catfish (*Pangasianodon hypophthalmus*) exploit food sources across anaerobic decomposition- and primary photosynthetic production-based food chains

Author(s):

Ayano Medo[1,*], Nobuhito Ohte[1], Hiroki Kajitani[1], Takashi Nose[1], Yuki Manabe[2], Tatsuya Sugawara[2], Yuji Onishi[3,8], Akiko S. Goto[3], Keisuke Koba[3], Nobuaki Arai[4], Yasushi Mitsunaga[5], Manabu Kume[4], Hideaki Nishizawa[1], Daichi Kojima[1], Ayako Yokoyama[2], Toshiro Yamanaka[6], Thavee Viputhanumas[7], & Hiromichi Mitamura[2,4]

The address in full of each author's primary affiliation:

[1]Graduate School of Informatics, Kyoto University, Yoshida-honmachi, Sakyo-ku, Kyoto, 606-8501 Japan

[2]Graduate School of Agriculture, Kyoto University, Kitashirakawa-Oiwake-cho, Sakyo-ku, Kyoto, 606-8502 Japan

[3]Center for Ecological Research, Kyoto University, 2-509-3 Hirano, Otsu, Shiga, 520-2113 Japan

[4]Field Science Education and Research Center, Kyoto University, Kitashirakawa-Oiwake-cho, Sakyo-ku, Kyoto, 606-8502 Japan

[5]Faculty of Agriculture, Kindai University, 3327-204 Nakamachi, Nara, 631-8505 Japan

[6]Department of Ocean and Environmental Sciences, Tokyo University of Marine Science and Technology, 4-5-7 Konan, Minato-ku, Tokyo 108-8477 Japan

[7]Inland Aquaculture Research and Development Division, Department of Fisheries, 50 Phahonyothin Rd., Lat Yao, Chatuchak, Bangkok, 10900 Thailand

[8]*Present Address*: Research Institute for Humanity and Nature, 457-4 Kamigamo-Motoyama, Kita-ku, Kyoto, 603-8047 Japan

*Corresponding author:

Ayano Medo

Postal address: Graduate School of Informatics, Kyoto University, Yoshida-honmachi, Sakyo-ku, Kyoto, 606-8501 Japan

E-mail (1): a-med@bre.soc.i.kyoto-u.ac.jp

E-mail (2): medo.ayano@gmail.com

Table S1. Stable sulfur isotope ratio ($\delta^{34}S$) and sulfur content of sediments in the Kaeng Krachan Reservoir in September 2019.

Station	Depth (m)	Acid-volatile sulfide		Total sulfur	
		$\delta^{34}S$ (‰)	Content (μmol S / g dry wt.)	$\delta^{34}S$ (‰)	Content (μmol S / g dry wt.)
1	23.2	–	ND	–	ND
2	24.8	+9.6	16.7	+8.4	19.6
3	27.5	+9.4	37.0	+7.9	89.7
4	30.0	+7.9	9.5	+6.3	51.6
5	15.5	+6.3	26.0	+6.0	94.4
6	30.0	+8.5	59.5	+8.0	85.9
7	3.0	–	ND	–	ND
9	6.0	–	ND	–	ND
10	5.5	–	ND	–	ND
11	3.4	–	ND	–	ND

ND: Not detected. Hyphen (–) indicates no measurements of $\delta^{34}S$.

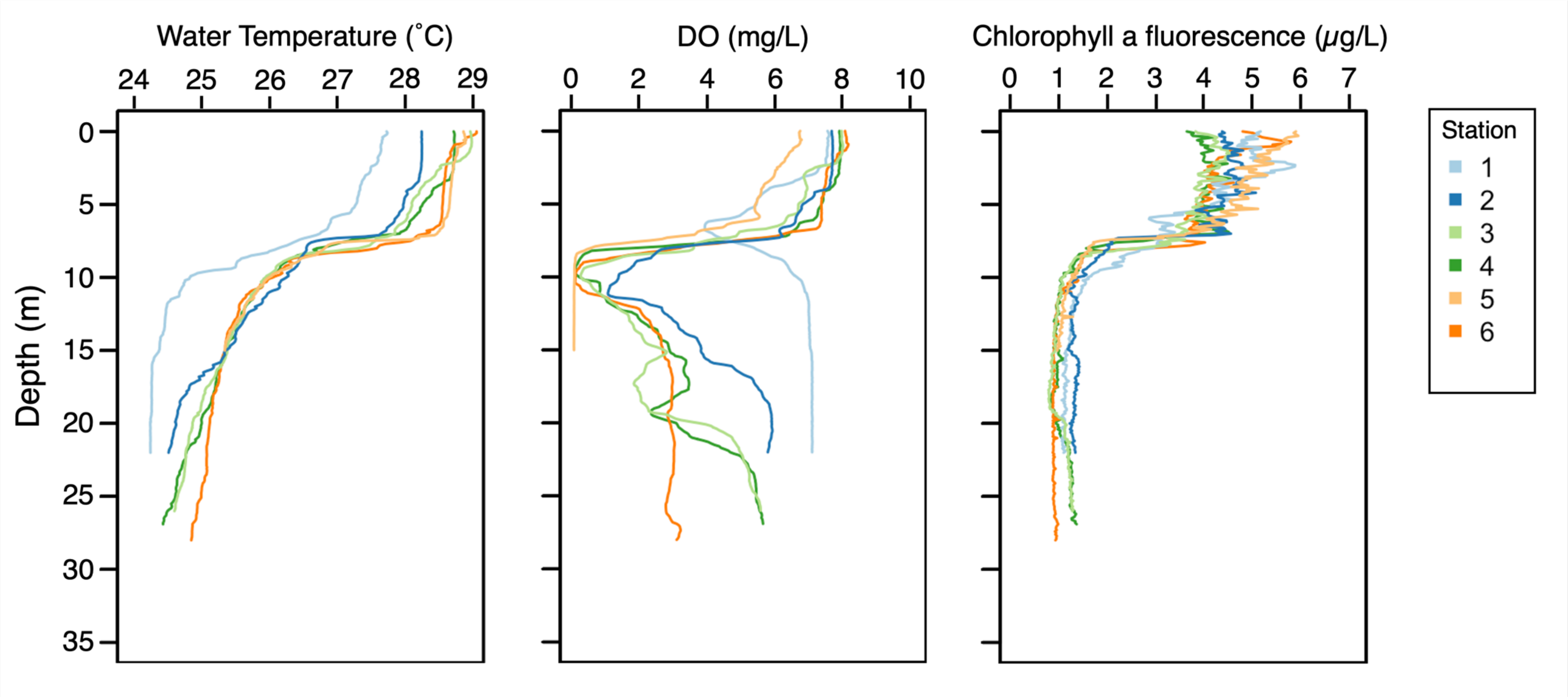

Figure S1. Vertical profiles of water temperature (ºC), dissolved oxygen (DO, mg/L), and chlorophyll a fluorescence (µg/L) at each location in the Kaeng Krachan Reservoir in September 2019.

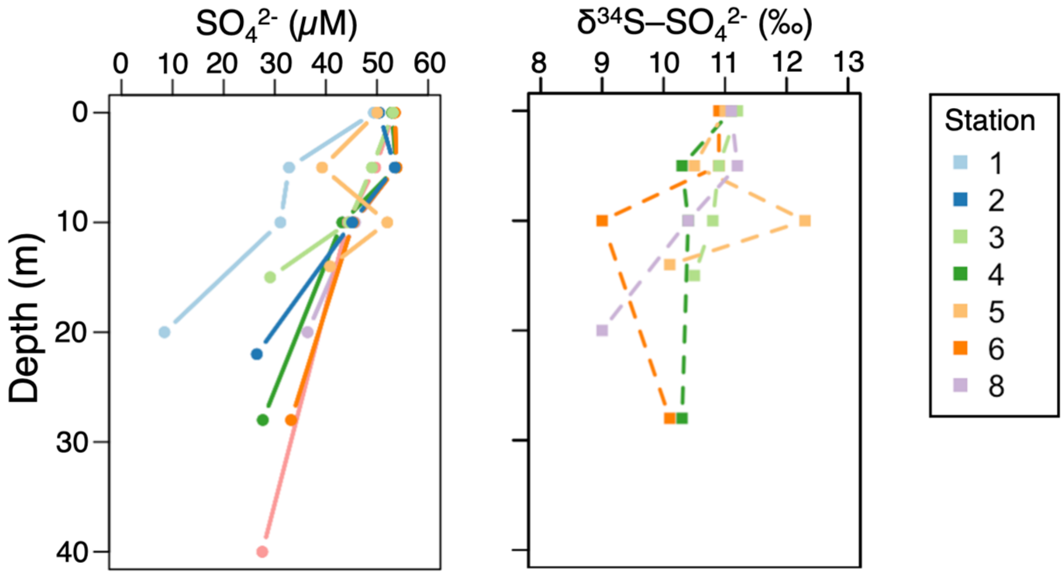

Figure S2. Vertical profiles of SO_4^{2-} concentrations and sulfate-stable sulfur isotope ratio ($\delta^{34}S_{sulfate}$) of column water at each location in the Kaeng Krachan Reservoir in September 2019.

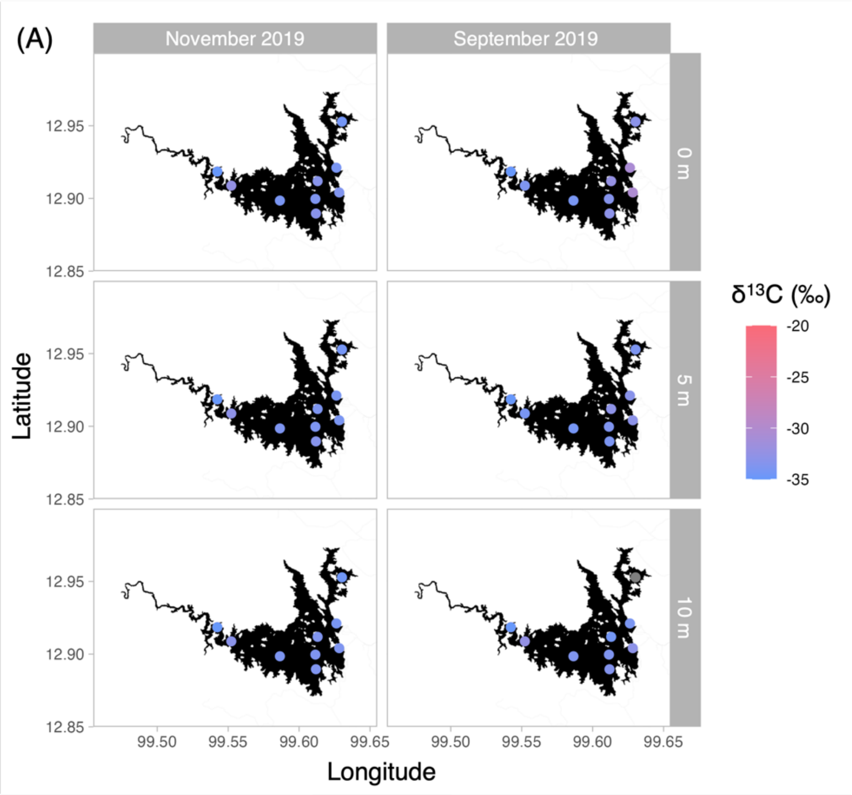
(A)
November 2019
September 2019
0 m
5 m
10 m
Latitude
Longitude
δ13C (‰)
-20
-25
-30
-35
12.95
12.90
12.85
99.50
99.55
99.60
99.65

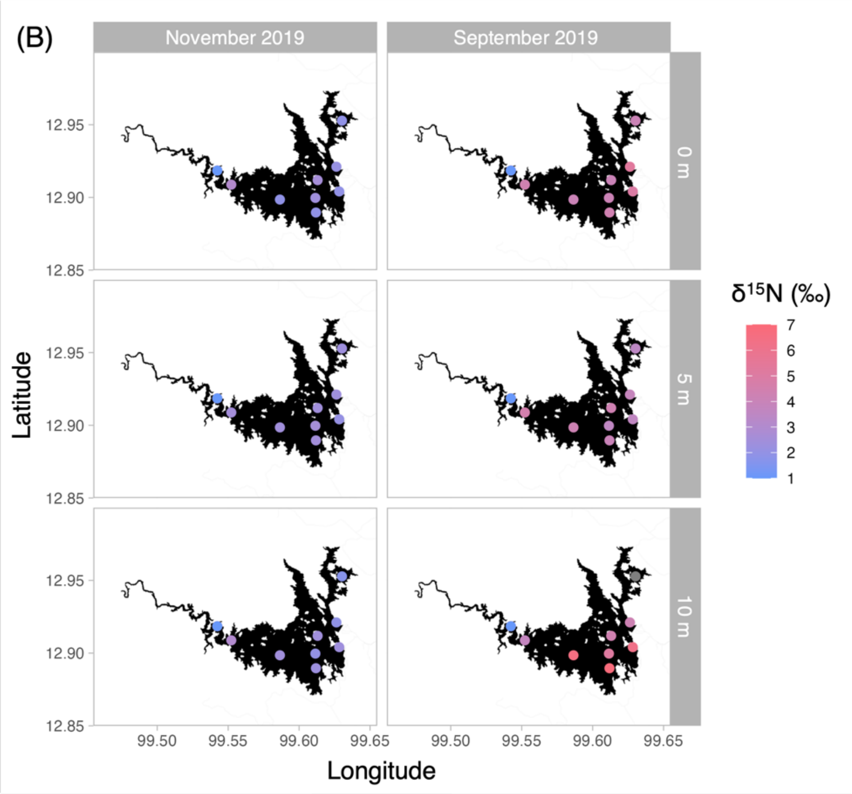
(B)
November 2019
September 2019
0 m
5 m
10 m
Latitude
Longitude
δ15N (‰)
7
6
5
4
3
2
1
12.95
12.90
12.85
99.50
99.55
99.60
99.65

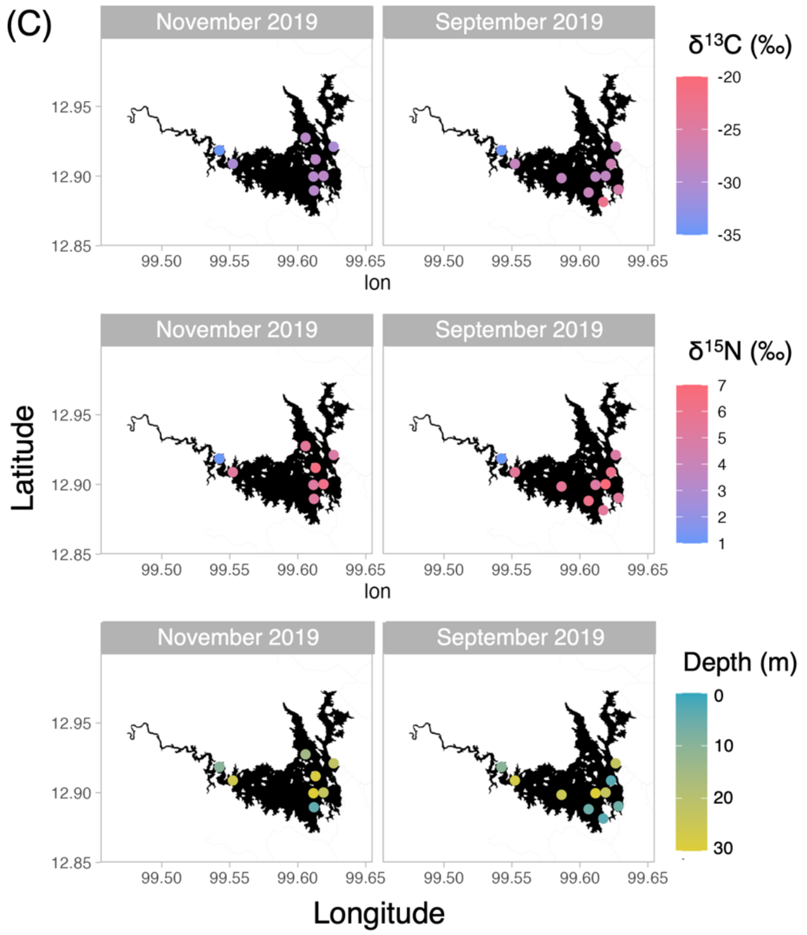

Figure S3. Horizontal and vertical variations of stable carbon and nitrogen isotope ratios ($\delta^{13}C$ and $\delta^{15}N$) in the Kaeng Krachan Reservoir. The variations of $\delta^{13}C$ (A) and $\delta^{15}N$ (B) in particulate organic matters. The variations of $\delta^{13}C$ and $\delta^{15}N$ in sediments (C). The all stable isotope data were cited from Medo et al., 2021.

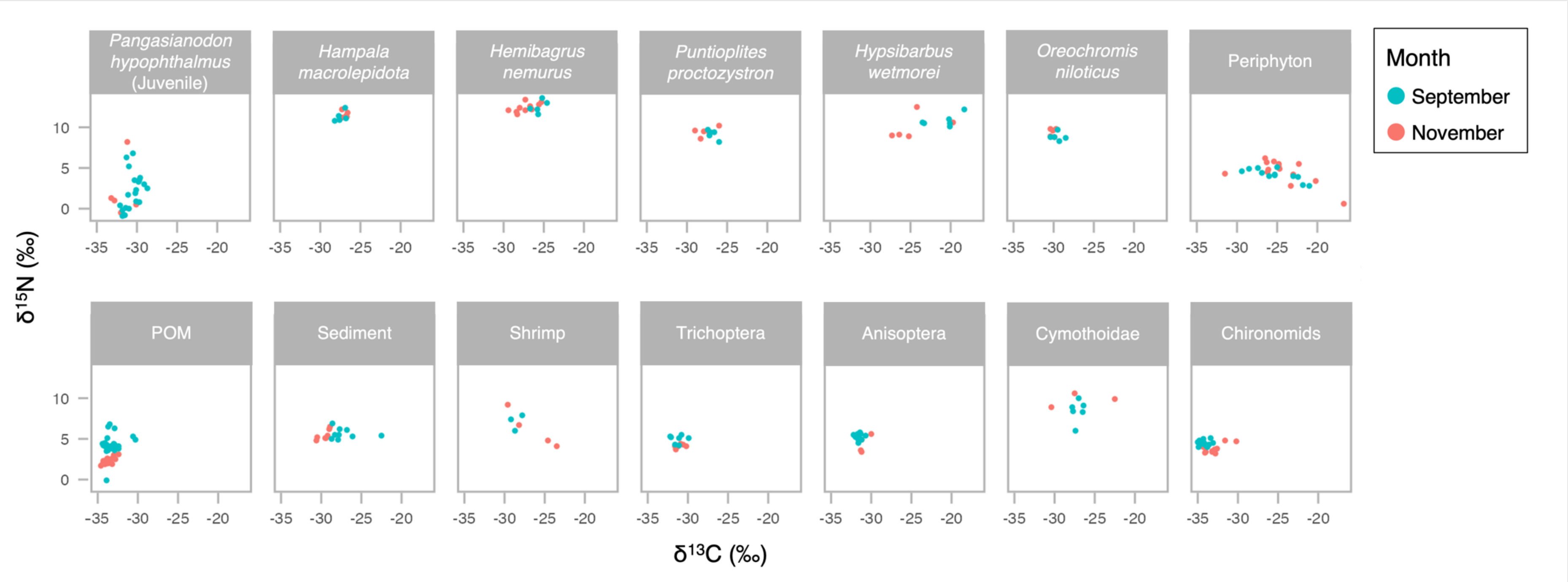

Figure S4. Monthly variations in stable carbon and nitrogen isotope ratios (δ^{13}C and δ^{15}N) of food web components in the Kaeng Krachan Reservoir in 2019. The stable isotope data except for the striped catfish *Pangasianodon hypophthalmus* were cited from Medo et al., 2021. Adult striped catfish was collected only in November–December 2019.
